# Fet-Net Algorithm for Automatic Detection of Fetal Orientation in Fetal MRI

**DOI:** 10.3390/bioengineering10020140

**Published:** 2023-01-20

**Authors:** Joshua Eisenstat, Matthias W. Wagner, Logi Vidarsson, Birgit Ertl-Wagner, Dafna Sussman

**Affiliations:** 1 Department of Electrical, Computer and Biomedical Engineering, Faculty of Engineering and Architectural Sciences, Toronto Metropolitan University, Toronto, ON M5G 1X8, Canada; 2 Division of Neuroradiology, The Hospital for Sick Children, Toronto, ON M5G 1X8, Canada; 3 Department of Diagnostic Imaging, The Hospital for Sick Children, Toronto, ON M5G 1X8, Canada; 4 Department of Medical Imaging, Faculty of Medicine, University of Toronto, Toronto, ON M5G 1X8, Canada; 5 Institute for Biomedical Engineering, Science and Technology (iBEST), Toronto Metropolitan University and St. Michael’s Hospital, Toronto, ON M5G 1X8, Canada; 6 Department of Obstetrics and Gynecology, Faculty of Medicine, University of Toronto, Toronto, ON M5G 1X8, Canada

**Keywords:** deep learning, fetal orientation, convolutional neural network, magnetic resonance image, architecture, fetal diagnosis

## Abstract

Identifying fetal orientation is essential for determining the mode of delivery and for sequence planning in fetal magnetic resonance imaging (MRI). This manuscript describes a deep learning algorithm named Fet-Net, composed of convolutional neural networks (CNNs), which allows for the automatic detection of fetal orientation from a two-dimensional (2D) MRI slice. The architecture consists of four convolutional layers, which feed into a simple artificial neural network. Compared with eleven other prominent CNNs (different versions of ResNet, VGG, Xception, and Inception), Fet-Net has fewer architectural layers and parameters. From 144 3D MRI datasets indicative of vertex, breech, oblique and transverse fetal orientations, 6120 2D MRI slices were extracted to train, validate and test Fet-Net. Despite its simpler architecture, Fet-Net demonstrated an average accuracy and F1 score of 97.68% and a loss of 0.06828 on the 6120 2D MRI slices during a 5-fold cross-validation experiment. This architecture outperformed all eleven prominent architectures (*p* < 0.05). An ablation study proved each component’s statistical significance and contribution to Fet-Net’s performance. Fet-Net demonstrated robustness in classification accuracy even when noise was introduced to the images, outperforming eight of the 11 prominent architectures. Fet-Net’s ability to automatically detect fetal orientation can profoundly decrease the time required for fetal MRI acquisition.

## 1. Introduction

In obstetric imaging, the position of the fetus in relation to maternal anatomical structures, such as the bladder and intestines, otherwise known as fetal presentation/orientation, must be determined [1]. The American Institute of Ultrasound in Medicine outlines fetal imaging protocols, stating that fetal presentation must be identified in all second and third-trimester fetal imaging examinations [2]. Four of the main fetal orientations are vertex (head down), breech (head up), oblique (diagonal), and transverse (sideways) [1,3,4]. Diagnosing fetal orientation is important for the choice of delivery [5,6,7].

Ultrasound (US) is the imaging method of choice to screen for fetal abnormalities. US uses sound waves to produce images [8]. However, recent advances in Magnetic Resonance Imaging (MRI) have demonstrated improved soft-tissue contrast and radiation-free properties and improved sensitivity over US imaging in some cases [9,10,11,12,13,14]. MRI uses strong magnetic fields, magnetic field gradients, and radio waves to form images [15,16]. As a result, fetal MRI is emerging as an essential imaging modality in evaluating complex fetal abnormalities and is even outperforming US in detecting many fetal pathologies [17,18,19,20]. The more accurate diagnoses from MRI, in turn, allow for better planning during the pregnancy for the postnatal and neonatal periods and even allow for possible fetal surgery where necessary [14,21]. MRI can also be used shortly before delivery for pelvimetric measurements to assess whether an attempt at vaginal delivery is feasible. While MRI is not the method of choice to determine fetal presentation, it is crucial to correctly assess fetal presentation when acquiring a fetal MRI to enable sequence planning. This can form the basis for more automated fetal MRI acquisition. In addition, fetal presentation needs to be correctly mentioned in the radiological report of the fetal MRI.

Machine learning is the application of computer algorithms that can self-learn through experience and data inputs [22]. Deep learning is a subfield within machine learning, which mimics human brain processes and uses large amounts of data and neural networks to learn patterns for recognition [22,23]. Deep learning, specifically convolutional neural networks (CNNs), have been previously applied to various fetal imaging applications, such as fetal head and abdomen measurement, artifact detection in 3D fetal MRI, and anomaly identification in a fetal brain, spine or heart [24,25,26].

For predicting the mode of delivery for a pregnancy, Kowsher et al. [27] applied 32 supervised machine and deep learning algorithms to a dataset consisting of 21 different metrics from 13,527 women [27]. The data metrics included age, blood circulation, and parity, among others [27]. Of all models tested, the Quadratic Discriminant Analysis algorithm yielded the best accuracy result at 97.9% [27]. In the case of deep learning algorithms, only a standard neural network has been tested to date, producing an accuracy of 95.4% [27]. Xu et al. [28] used CNNs to create heatmaps that can then estimate the fetal pose of a 3D fetal MRI volume with a Markov Random Field model. Xu’s work provides an estimation of fetal pose in space, which is important for monitoring fetal movement. Nevertheless, this approach does not provide a classification of the fetal presentation with respect to the maternal anatomy using internationally acceptable classes (vertex, breech, oblique, and transverse) [29]. To the best of our knowledge, such an approach of applying CNNs to automatically detect fetal orientation, with the motivation of facilitating delivery planning and fetal MRI acquisition, has not been performed, which is the goal of our study. We will then compare the performance of our novel CNN algorithm with those of other state-of-the-art CNN architectures, including VGG, ResNet, Xception and Inception.

## 2. Materials and Methods

### 2.1. Dataset

**Figure 1:**
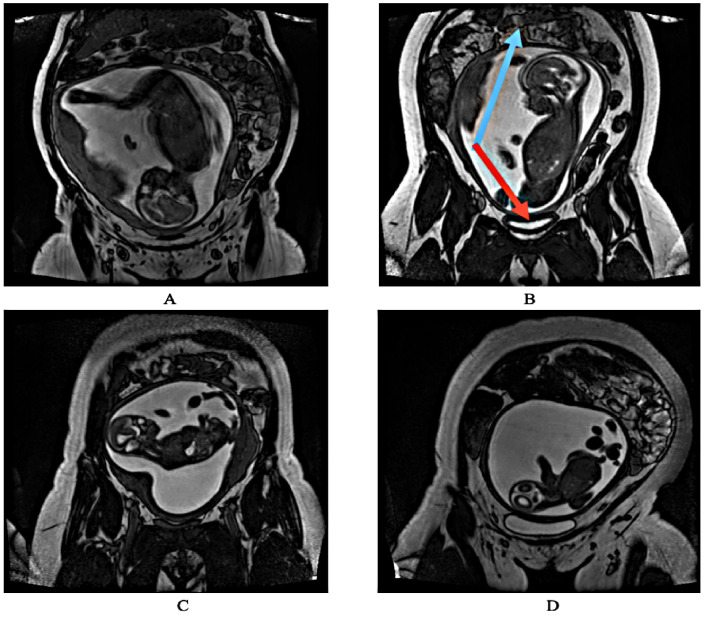
The four different labels of fetal orientation. (**A**) Vertex (head down). (**B**) Breech (head up). The blue arrow is pointing to the maternal intestines. The red arrow is pointing to the maternal bladder. These two maternal organs were used as reference while manually labeling each two-dimensional (2D) slice based on the fetal orientation. (**C**) Transverse (sideways). (**D**) Oblique (diagonal).

This study used 144 T2-weighted coronal three-dimensional (3D) fetal MRI datasets, which were acquired between 2008 and 2021. The spatial resolutions of the 2D MRI slices from each 3D MRI dataset were inconsistent, as some were 512 × 512, while others were 256 × 256. This inconsistency was not of serious concern, and so no up/downsampling of the images was conducted. The 3D fetal MRIs were acquired on a 1.5T or 3.0T (Magnetom Avanto, Avanto-fit or Skyra, Siemens Healthineers, Erlangen, Germany) using a True Fast Imaging with Steady State Free Precession (TrueFISP) sequence. In order to conduct supervised learning, the fetal MRI datasets were labeled prior to training, validating and testing Fet-Net. The distribution of fetal orientations among the 144 3D MRIs is as follows: 65 were vertex, 61 were breech, 11 were transverse, and 7 were oblique. Figure 1 illustrates how all the 2D MRI slices were classified according to their respective fetal presentations. The number of 2D MRI slices in the 144 3D MRI datasets roughly ranged from 52 to 120. The gestational ages (GA) of the fetal MRIs range from 20 + 2 weeks to 38 + 1 weeks.

Data augmentation was performed on the oblique and transverse 2D slices to offset the lower natural prevalence of transverse and oblique orientations that were found in our available dataset. This data augmentation ensured an even distribution of images for training, validation and testing. Various rotation, zooming, shearing, and both vertical and horizontal flipping operations were performed on the transverse and oblique 2D slices to generate additional samples. This led to a consistent tally of 1530 2D MRI slices for the four labels. Therefore, there was a total of 6120 2D MRI slices. The balance in the number of 2D MRI slices for all four labels was significant, as it ruled out Fet-Net underperforming due to inherent biases toward a specific label with more samples. The 2D images for each label were divided using a 56–24–20% split for training, validation, and testing, respectively. It is typically common practice to have a dataset split of 80–10–10% for training, validation and testing, respectively [30]. Having larger testing and validation sets removes more instances for the predictive model to learn; yet, it provides more confidence in the model’s results, as there is less likelihood that the given results are obtained by chance and demonstrates a model’s robustness. Such an approach still showed potential for high-level classification by Madaan et al. (2021) [31]. An example of each label is illustrated in Figure 1.

The fetal MRIs were provided as grayscale images; however, since the prominent architectures such as ResNet and VGG could only accept image inputs in the RGB (Red–Green–Blue) color scale, all images were pre-processed and converted from grayscale to RGB. This was performed to ensure consistency in the images being presented to each CNN. Every image was resized to 120 × 120 pixels and normalized by dividing all pixels by 255, bringing the range of pixels in a given image from 0–255 to 0–1.

### 2.2. Architecture

In a study conducted by Haque et al. [32] to detect COVID-19 based on chest X-ray images, three novel CNN architectures were tested against three different pre-trained CNN architecture models: ResNet50, VGG16, and VGG19. All three novel CNN architectures with accuracies of 97.9%, 91.8%, and 95.0% performed better than the three pre-trained architectures, with accuracies of 88.3%, 79.6%, and 62.2% for ResNet50, VGG16, and VGG19, respectively [32]. This study demonstrated the improved effectiveness of a novel architecture with fewer layers over the increased number of layers seen in the ResNet50, VGG16, and VGG19 architectures for this specific application [32].

**Figure 2:**
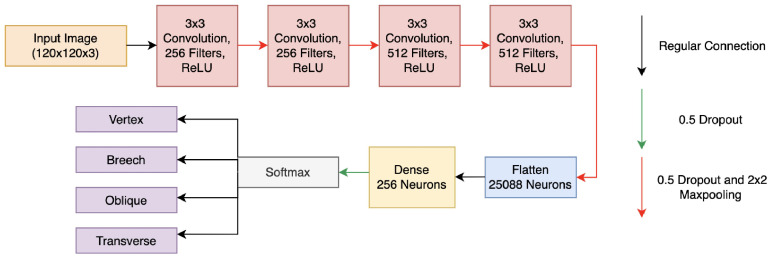
Complete Fet-Net architecture and classification of a fetal MRI.

The Fet-Net architecture, which follows a sequential CNN approach inspired by the approach presented by Haque et al. [32], is illustrated in Figure 2:

There were seven total layers in Fet-Net. Fet-Net’s feature extraction portion consisted of four convolutional layers, in which there were 2D convolutional filters and a 2D 2 × 2 MaxPooling operation. The first two convolutional layers have 256 3 × 3 filters, whereas the following two have 512 3 × 3 filters. The purpose of the MaxPooling layers was to reduce the dimensionality of the images, which emphasizes the pixels with the essential information. An ascending number of convolutional filters was used in the feature extraction portion of the architecture [32]. ReLu activation functions were in every layer of the architecture to add non-linear properties to the predictive model, as the convolution operation is a linear process. This is a necessary step, as the process of image classification is inherently a non-linear operation [33]. In order to prevent overfitting of the predictive model to the training images, dropout rates of 0.5 were added to each layer of the network. The extracted features were then compiled into one fully connected layer to begin classification. The neural network had three layers: more specifically, a fully connected layer of 25,088 neurons, one hidden dense layer of 256 neurons, and then the final layer had four neurons representing the four labels for classification of fetal orientation. The final classification depended on the neuron with the highest probability based on the softmax function, which was used for multi-class classification.

### 2.3. Experiment

#### 2.3.1. Experimentation Set-Up

The fetal orientation classification algorithm was written in Google Colaboratory (Colab), an environment for coding in Tensorflow, which allows for training machine learning algorithms in Python [34]. Keras, an open-source library compatible with Tensorflow, was used for constructing the architecture of Fet-Net [34]. Google Colab-pro uses one of the NVIDIA-T4 or NVIDIA-P100 Graphic Processing Units (GPU).

#### 2.3.2. Five-Fold Cross-Validation Experiment

The Adam optimizer was chosen for the model’s training, as it converges quicker than the generic stochastic gradient descent (SGD) algorithm due to its adaptive learning rate property [35]. This more rapid convergence can potentially lead to improved results. The sparse categorical cross-entropy loss function was used, as the labels were not one-hot encoded. The softmax activation function was used in the last step before classification, as it provides a probability for each label. Whichever neuron has the highest probability becomes selected for the classification. The softmax activation function is applicable for multi-label classification problems. Fet-Net’s main experimentation process was performed using a 5-fold cross-validation technique. Cross-validation provides more confidence in the algorithm’s results, as the chances of skewed results by an arbitrary seed are reduced. Nevertheless, it was established at the beginning of the algorithm that there be no variations in the seeds to ensure that the changes in results were due to either a change in architecture or hyperparameter rather than a change in the seed. Every image in the dataset was used for testing, since 5-fold cross-validation uses a different 20% of the total dataset for each fold until every image is tested. The results presented in the next section for the novel architecture are those obtained using a learning rate of 5 × 10${}^{-4}$, a batch size of 64, 75 epochs and dropout rates of 0.5.

The objective of this experiment was to build a CNN architecture that outperformed prominent CNN architectures. Therefore, hyperparameter optimization was conducted for Fet-Net and for its architecture, and this process was repeated for VGG16, VGG19, ResNet50, ResNet50-V2, ResNet101, ResNet101-V2, ResNet152, ResNet152-V2, Xception, Inception-Resnet-V2, and InceptionV3. One-sided ANOVA statistical testing with a 0.05 level of significance was performed to determine whether the differences in results were statistically significant.

#### 2.3.3. Performance Metrics

Performance metrics, such as accuracy, precision, recall and F1-score were used to analyze the performance of the trained Fet-Net architecture. Accuracy was defined as how many total images from the testing set were correctly categorized among the four labels [34]. The recall for a given label was the number of correct predictions made of the true number of observations for the given label [34]. Precision was the number of correct predictions out of all the predictions made for a given label. The F1 score is a more informative measure of the model’s performance for each specific label, as it is a weighted average of the label’s recall and precision [34]. The four metrics were calculated as follows [34]:

(1) $$Accuracy=\frac{1}{n}\sum_{i=1}^{n}\left(Ytrue_{i}=Ypred_{i}\right)\;$$

In Equation (1), *n* represents the number of testing images, *Ytrue_i_* is the actual label of the test image, and *Ypred_i_* is what the predictive model predicts for the image’s label. Therefore, the accuracy was determined by the number of times the predictive model predicted the true label over the number of testing images.

(2) $$Precision=\frac{TP}{TP+FP}$$

(3) $$Recall=\frac{TP}{TP+FN}$$

(4) $$F1\;-\;Score=\frac{2(Precision\times Recall)}{Precision+Recall}$$

In Equations (2) and (3), *TP* is true positives, *FP* is false positives, and *FN* is false negatives. Beyond the four metrics mentioned above, the model’s loss was used to determine how well the model was learning. A low loss indicates high confidence in the model’s predictions. Accuracy and loss curves were plotted for every fold each time the algorithm was run. These were inspected to ensure no overfitting and that proper learning occurred through steadily increasing accuracy curves to a plateau and steadily decreasing loss curves to a steady minimum for both training and validation.

#### 2.3.4. Validation Experiment

A validation experiment was performed with a new holdout testing set. With a 5-fold cross-validation experiment, we could not control whether 2D MRI slices from the same 3D MRI dataset were used in both training and testing. Therefore, to validate the performance of Fet-Net, 605 2D MRI slices were used for holdout testing, which comprises nearly 10% of the entire dataset [30]. It was ensured that no 2D MRI slices from a 3D MRI dataset that was used for training were used in the holdout testing set. Fet-Net and all eleven prominent architectures were tested with the 605 2D MRI slices.

#### 2.3.5. Ablation Study

An ablation study was conducted to investigate the contribution of each component of the Fet-Net architecture to its performance. This entailed removing a single layer or architecture component at a time, running the algorithm with the new architecture, and recording/inspecting the new accuracy, precision, recall, F1-score, loss, and learning curves.

#### 2.3.6. Signal-to-Noise Ratio Test

Lastly, we conducted a test to investigate Fet-Net’s ability to correctly classify the fetal orientation of a 2D MRI with a relatively lower signal to noise ratio (SNR). To perform this, 60 2D slices from each label were removed for the purpose of holdout testing. Gaussian white noise with a mean of 0.1 and variance of 0.01 was added to all 60 2D MRI slices of all four labels, thereby reducing the SNR of each image. This then allowed for an observation of how a model trained on undistorted images performed on unseen distorted/noisy images.

## 3. Results

### 3.1. Fet-Net Results

Fet-Net classified the fetal orientation of the 6120 2D MRI slices with an average accuracy of 97.68% and loss of 0.06828 during the 5-fold-cross-validation experiment. The three remaining performance metrics (average precision, recall and F1-score) of the Fet-Net architecture for each individual label are summarized in Table 1.

### 3.2. Comparison of Architectures

The average testing accuracy and loss across the five testing folds for all the various architectures are summarized in Table 2. It should be mentioned that the average precision, recall and F1-score across all five folds often were the same value as the average accuracy for the respective architecture. The number of parameters for the prominent architectures are reported for when personalized input and output layers in the network were configured.

**Figure 3:**
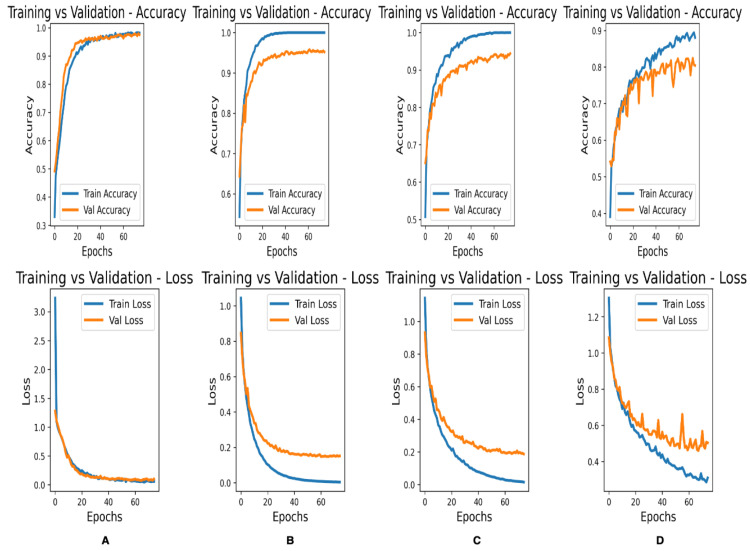
Accuracy and loss curves for training and validation of the second data-fold for various CNN architectures. (**A**) Fet-Net architecture. (**B**) VGG-16. (**C**) VGG19. (**D**) ResNet-50.

Figure 3 shows the accuracy and loss curves for the training and validation of the complete Fet-Net architecture and three of the prominent architectures:

### 3.3. Validation Experiment of Fet-Net

A holdout testing set was created in order to validate the promising results of the 5-fold cross-validation experiment. The accuracy and loss values of Fet-Net and all eleven prominent architectures are illustrated in Table 3.

### 3.4. Ablation Study

**Figure 4:**
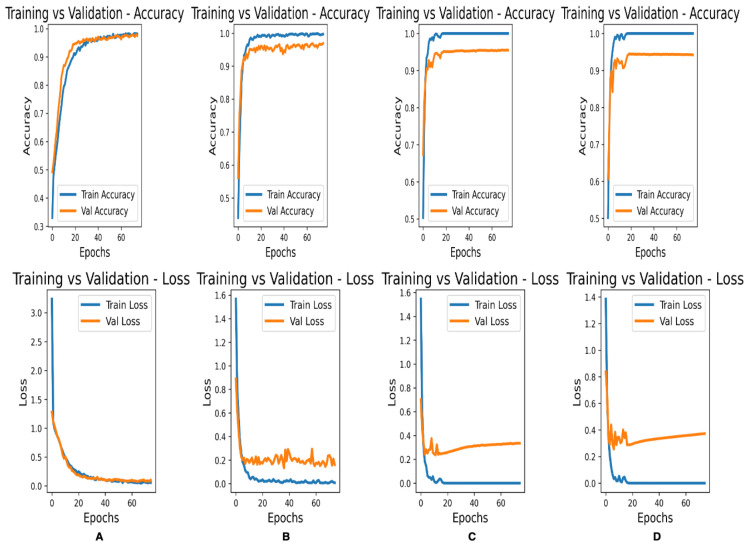
Accuracy and Loss curves for training and validation of the second data-fold for various CNN architectures. (**A**) Complete Fet-Net architecture. (**B**) No dropout in the feature extraction component. (**C**) No dropout in the feature extraction component or classification component. (**D**) No dropout in the feature extraction component or classification component, and no hidden dense layer of 256 neurons.

For the ablation study, architecture components were removed one after another, starting with the dropouts, then the hidden dense layer, and finally proceeding to remove the convolutional layers [36]. The average testing accuracy and loss across the five testing folds were recorded for all the various ablated architectures. Again, it should be mentioned that the average precision, recall and F1-score across all five folds often were the same value as the average accuracy for the respective architecture. The results are displayed in Table 4:

Figure 4 shows the accuracy and loss curves for the training and validation of the complete Fet-Net architecture and three of the ablated architectures:

### 3.5. Testing on Noisy Images

Table 5 demonstrates the results of the different architectures on noisy images. Only the testing images were given additional Gaussian white noise, and so the testing images had a lower SNR than those used for the model’s training. Fet-Net demonstrated a reduced accuracy of 74.58%; nonetheless, this was expected given the noisy testing images.

## 4. Discussion

Fet-Net, the seven-layer convolutional neural network, demonstrated an average accuracy and F1-score of 97.68% and a loss of 0.06828 in classifying the fetal orientation of 6120 2D MRI slices during a 5-fold cross-validaton experiment. Fet-Net accurately classified the 2D MRI slices as one of vertex, breech, oblique or transverse with precision and recall scores above 95.23% for all four individual labels.

It is interesting to note that Fet-Net has only 10,556,420 parameters in its architecture, whereas out of the prominent networks, the next closest in terms of number of parameters is VGG16 with 14,847,04. The architecture with the highest number of parameters is ResNet152, with 58,896,516. During transfer learning, the transferred layers were frozen, which yielded better results than when the layers were not frozen. Therefore, the comparison between Fet-Net and the transfer learning models is when the layers were not trainable for the prominent CNNs. The number of parameters needed for a classification task depends on the dataset being used. Certain datasets may require a denser network to be able to classify correctly. Nevertheless, if a network is too complex for a more straightforward classification task, then trends such as overfitting will be illustrated. This was apparent in looking at the curves of Figure 3B–D, as the validation curves begin to diverge from the training curves rather than converge. Therefore, despite the fewer parameters, Fet-Net’s architecture performed better than the more complex architectures with far more parameters. Data normalization was an essential operation in the algorithm, since it markedly improved the loss of the model and increased the speed of convergence during the model’s training. Without normalization, the average accuracy decreased to 59.53%, while the average loss increased to 0.81983. This is evidence of a model that is unable to learn with each successive epoch when normalization is not performed.

VGG16 performed the best after Fet-Net, with an accuracy of 96.72% and a loss of 0.12316. This accuracy was lower than Fet-Net by 0.96%, and the loss was greater by 0.05488. The most drastic difference in performance was demonstrated by ResNet101, in which the accuracy was lower by 15.56%, and the loss was greater by 0.40258. One-sided ANOVA testing was used to determine the statistical difference between Fet-Net’s results and those of the prominent CNN architectures. Based on the ANOVA test, the difference in results of VGG16 from Fet-Net was statistically significant (*p* < 0.05). Therefore, Fet-Net outperformed all 11 prominent architectures in a significant manner. Fet-Net’s precision and recall of all four individual labels outperformed all other architectures, apart from VGG16’s precision for the transverse label by 0.3742% and VGG16’s recall for the oblique label by 0.3936%. Fet-Net’s lower loss than all other architectures demonstrates its ability to predict the correct fetal orientation with higher confidence. In other words, on average, the neuron of the correct label in the last layer of the artificial neural network has a higher probability associated with its classification than when the other prominent CNN architectures are tested.

Accuracy and loss curves are very telling regarding how well a model has learned. The curves illustrate whether overfitting or underfitting have occurred depending on the resemblance of the validation curve to the training curve. It is essential to have the training and validation accuracy curves gradually increase together to a consistent plateau and loss curves that slowly decline together to a consistent minimum. Figure 3 demonstrated that only Fet-Net (Figure 3A) follows the desired trend, whereas all other architectures demonstrated overfitting, as the plateau of the validation accuracy curves were below the training accuracy curves, and the validation loss curves were above the training loss curves.

Despite the decreased accuracy and increased loss for Fet-Net in Table 3, the results of the validation experiment were still encouraging. It was expected that Fet-Net would not perform as well with the 605 unseen 2D MRI slices, as none of the slices from these 3D MRI datasets were used in training. During this test, five-fold cross-validation was not performed; thus, three different random seeds were set to ensure that the results for Fet-Net were not obtained by chance. Fet-Net’s average F1-scores across the three seeds for all four labels were above 80%. Fet-Net outperformed all other architectures by at least 5.34%, and its loss was lower by at least 0.5694.

The results of the ablation study were also encouraging, as they demonstrated the role each component of the Fet-Net architecture plays in achieving the obtained performance. First, as a test, L1 and L2 regularization were separately added to the architecture to see if it improved performance; yet, it significantly increased the loss and was therefore left out of the architecture. The Adam optimizer has advantages over the SGD optimizer due to its adaptive learning rate. However, Zhang argues that predictive models with the SGD optimizer can generalize more effectively than those with the Adam optimizer, which is an essential property of an image classifier [35]. Both optimizers were tested during the testing phase of the experiment, and it was apparent that the SGD optimizer failed to converge, as the accuracy and loss values did not improve during the training process, which ultimately led to poor generalization. Therefore, the Adam optimizer was used. Typically, dropouts are only used in the neural network component of the architecture; however, as shown in Table 4, the results were enhanced by adding the dropout components into the feature extraction portion of the architecture. The dropout rate was set to 0.5 across the entire architecture, which is typically the limit for dropout rates. The accuracy and loss metrics were negatively impacted by dropout rates below 0.5. The 0.5 dropout rate prevented overfitting, which became apparent even when a 0.4 dropout rate was used. From Table 4, it is clear how the accuracy of Fet-Net was reduced with each successive ablation. From the complete architecture to its most ablated version, the accuracy was reduced by 18.84%, and the loss increased by 1.0489. The ANOVA test demonstrates the statistical significance of the difference in accuracy and loss between the complete architecture and the fully ablated architecture with *p*-values of 8.8310 × 10${}^{-10}$ and 2.2410 × 10${}^{-11}$ for the accuracy and the loss, respectively. Statistical testing was conducted for the accuracies between every successive row of Table 4, and it was found that the differences in accuracies were all significant (*p*-values < 0.05) except for the removal of the hidden layer of 256 neurons after removing the dropouts from the entire architecture. However, the hidden layer was still incorporated into the architecture, as the difference in loss between the complete architecture and an ablated version with just the hidden layer removed is statistically significant (*p*-value = 0.004). Figure 4 illustrates a more pronounced overfitting trend as more components are removed from the architecture.

Fet-Net again outperformed all other prominent architectures, with the exception of the ResNet152 architecture for loss. As the magnetic field strength of an MRI increases, so too does the signal-to-noise ratio [37]. The signal-to-noise ratio also increases with the repetition time of an MRI, which is the amount of elapsed time between two pulse sequences on one slice [37]. If a model with fewer parameters performs better on images of reduced SNR, then the amount of time for MRI acquisition does not need to be as long. The differences in accuracy between Table 2 and Table 5 demonstrate how most of the prominent architectures are much more affected by noisier images. For example, the VGG16 architecture only trailed Fet-Net by 0.96% for accuracy on undistorted images. However, when tested with noisy images, the VGG16 architecture trailed Fet-Net by 7.08%. The current accuracy of 74.58% for Fet-Net is not satisfactory; however, these results illustrate that a model with fewer parameters is not as affected by lower SNR MRIs. With this information, it may be possible to conduct MRIs faster and build an optimized predictive model with fewer parameters than the current prominent architectures, which could ultimately help in alleviating the healthcare systems pertaining to the backlog in MRI appointments.

There are some limitations associated with the experimental process. Despite treating the 2D image slices as independent after performing data augmentation, there is an inherent correlation between the images of one fetus. In addition, the 2D image slices of one 3D fetal MRI have subtle differences; yet, they still resemble one another. While there were no direct duplicates of 2D image slices between the training and validation/testing sets, the randomized split of 2D slices across training/validation/testing sets during the 5-fold cross-validation experiment may give rise to images in the validation/testing sets resembling an image in the training set. We, therefore, performed a validation experiment to provide a more informative representation of Fet-Net’s capabilities. Additionally, this study uses one dataset of 6120 2D MRI slices split into five separate training validation and testing folds; however, further comparisons of deep learning algorithms using different MRI datasets should be conducted to confirm these promising results.

## 5. Conclusions

In this experiment, we created a novel CNN architecture, which despite having fewer layers and parameters than eleven prominent CNN architectures still provided improved classification accuracies in terms of the fetal orientation in 6120 2D MRI slices. The training process shown by the curves of Fet-Net followed those of the desired training curves previously explained and more than those of the prominent architectures. All components of the Fet-Net architecture significantly contributed to its improved performance. Introducing an automated approach for detecting fetal orientation can significantly impact the MRI acquisition process. Fet-Net’s impact on sequence planning for fetal MRI acquisition may allow for expedited MRI acquisition and reduce the number of required MRI operators. Future work on this project would entail adding subclassifications within all four labels, such as the specific direction the fetus is facing (left or right).

## Figures and Tables

**Table 1:** Summary of performance metrics for Fet-Net during the 5-fold cross-validation experiment.

	Vertex	Breech	Oblique	Transverse
Average Precision (%)	99.35	99.35	96.12	95.87
Average Recall (%)	99.93	99.80	95.23	95.75
Average F1-Score (%)	99.64	99.57	95.67	95.81

**Table 2:** Summary of performance metrics for Fet-Net and prominent CNN architectures during the 5-fold cross-validation experiment.

Architecture	Average Accuracy (%)	Average Loss	Number of Parameters
Fet-Net	97.68	0.06828	10,556,420
VGG16	96.72	0.12316	14,847,044
VGG19	95.83	0.15412	20,156,740
ResNet-50	88.37	0.40604	24,113,284
ResNet-50V2	95.20	0.16328	24,090,372
ResNet-101	82.12	0.47086	43,183,748
ResNet-101V2	94.69	0.18866	43,152,132
ResNet-152	84.12	0.41756	58,896,516
ResNet-152V2	94.61	0.20502	58,857,220
Inception-ResnetV2	94.20	0.21042	54,731,236
InceptionV3	93.83	0.21720	22,328,356
Xception	96.08	0.13956	21,387,052

**Table 3:** Summary of performance metrics of Fet-Net and prominent CNN architectures during the validation experiment.

Architecture	Accuracy (%)	Loss	Number of Parameters
Fet-Net (Average of 3 Seeds)	82.20	0.4777	10,556,420
VGG16	63.80	1.6586	14,847,044
VGG19	61.82	1.6588	20,156,740
ResNet-50	53.06	1.7716	24,113,284
ResNet-50V2	70.58	1.1676	24,090,372
ResNet-101	57.85	1.3354	43,183,748
ResNet-101V2	66.12	1.4789	43,152,132
ResNet-152	60.00	1.2846	58,896,516
ResNet-152V2	76.86	1.0471	58,857,220
Inception-ResNetV2	63.64	1.5332	54,731,236
InceptionV3	59.17	1.7725	22,328,356
Xception	62.48	1.5365	21,387,052

**Table 4:** Summary of performance metrics of Fet-Net during the ablation study.

Component(s) Removed (Sequentially)	Average Accuracy (%)	Average Loss	Number of Parameters
Full Architecture	97.68	0.06828	10,556,420
Dropout in Feature Extraction Section	96.58	0.1614	10,561,028
Dropout in Feature Extraction and Classification Sections	94.97	0.30464	10,561,028
Dense Layer with 256 Neurons	94.51	0.28262	4,237,572
Second Convolutional Layer with 512 Filters	91.70	0.54048	2,238,212
First Convolutional Layer with 512 Filters	88.38	0.92238	1,518,852
Second Convolutional Layer with 256 Filters	87.11	0.75504	3,693,572
First Convolutional Layer with 256 Filters (1 filter left for functional purposes)	78.84	1.11718	14,432

**Table 5:** Summary of performance metrics of Fet-Net and prominent CNN architectures with a decreased SNR testing dataset.

Architecture	Accuracy (%)	Loss
Fet-Net	74.58	0.7491
VGG16	67.50	1.4996
VGG19	49.58	2.8058
ResNet-50	69.58	0.8897
ResNet-50V2	55.83	2.0550
ResNet-101	60.00	0.9695
ResNet-101V2	61.25	2.6991
ResNet-152	70.83	0.7185
ResNet-152V2	57.50	2.3606
Inception-Resnet-V2	66.67	1.2373
InceptionV3	56.57	2.6833
Xception	62.92	1.9649

## Data Availability

The datasets presented in this article are not readily available because the hospital’s research ethics board does not permit sharing clinical images. Requests to access the datasets should be directed to DS, dafna.sussman@torontomu.ca.

## Abbreviations

The following abbreviations are used in this manuscript:

2D	Two-Dimensional
3D	Three-Dimensional
CNN	Convolutional Neural Network
MRI	Magnetic Resonance Imaging
SNR	Signal-to-Noise Ratio
US	Ultrasound
